# Depressive symptoms and their association with social determinants and chronic diseases in middle-aged and elderly Chinese people

**DOI:** 10.1038/s41598-018-22175-2

**Published:** 2018-03-01

**Authors:** Qiaolan Liu, Hui Cai, Lawrence H. Yang, Yong-Bing Xiang, Gong Yang, Honglan Li, Yu-Tang Gao, Wei Zheng, Ezra Susser, Xiao-Ou Shu

**Affiliations:** 1 Division of Epidemiology, Department of Medicine, Vanderbilt Epidemiology Center, Vanderbilt-Ingram Cancer Center, Vanderbilt University School of Medicine, Nashville, TN United States; 2 West China School of Public Health, Sichuan University, Chengdu, Sichuan China; 3 Department of Epidemiology, Mailman School of Public Health, Columbia University, New York, NY United States; 4 Department of Social and Behavioral Sciences, College of Global Public Health, New York University, New York, NY United States; 5 Department of Epidemiology, Cancer Institute of Shanghai Jiao Tong University, Shanghai Cancer Institute, Shanghai, China

## Abstract

Depression is one of the most prevalent mental disorders worldwide. Little information is available regarding association of depressive symptoms (DS) with cancer and chronic diseases among middle-aged and elderly Chinese in a population-based setting. In this study we evaluated the prevalence and examined correlates of DS in two population-based cohort studies. Included in the analyses were 103,595 people with a mean age of 61.8 years at the DS assessment. The prevalence of DS was 2.4% in men and 5.6% in women. We found elderly participants, those with lower BMI, or chronic diseases were more likely to experience DS. Having a history of stroke (odds ratio (OR) = 2.2 in men and 1.8 in women), cancer (OR = 3.3 in men and 1.9 in women), or Parkinson’s disease (OR = 3.1 in men and 2.7 in women) was associated with high DS. In women, high income and high education levels were inversely related to DS. Being a single woman, long-term or heavy female smoker was associated with high prevalence of DS. High BMI was correlated with low prevalence of depression in men. Our data suggests a low prevalence of DS among middle-aged and elderly people in Shanghai, China. Age, education, income, marital status, smoking, BMI, and certain health conditions were associated with DS.

## Introduction

Depression, characterized by symptoms of sadness, depressed mood, and loss of interest, is one of the most prevalent disorders worldwide and accounts for 5.9% of total all-cause disability-adjusted life years (DALYs)^1^. Depression is associated with poor quality of life, prevalence of cancer, chronic diseases and suicide, and thus may contribute to an increase in mortality^2^. Depression casts a heavy burden on families, communities and health services in both developed and developing countries^3,4^.

The prevalence of depressive symptoms (DS) varies widely, from 1% to 16% among middle-aged and elderly people in studies conducted in Western countries^5–7^. One study reported DS prevalence of elderly Chinese people being 3.9% during 1980s and 1990s^8^. Two recent meta-analysis studies reported the prevalence has been increasing rapidly because of urbanization and environmental changes in China, up to 17% among people aged 55 years and above using the Center for Epidemiologic Studies Depression Scale (CES-D)^9,10^.

Several socio-demographic characteristics, such as age, gender, marital status, education level, and income have been associated with DS^11^, although the evidence is not entirely consistent^7,10^. Age is associated with DS in many studies^12^, and interestingly, this association is inverse or U-shaped in several studies^13–15^. Low socioeconomic status was found to be associated with severe DS in both men and women and higher education level was related with lower risk of DS^16–18^. However, few studies have reported no association between income and DS^19^ as well as between education level and DS^11,15^. Research on the relationship between DS and lifestyles, such as smoking, drinking and BMI, to date has been scarce in mainland China. Inconsistent data with respect to lifestyle factors and DS can be found in many studies in the other countries^20–22^ and could be due to different study settings, races, different depression assessment scale, and cultures of participants.

Depressive symptoms have been found to be related to cancer and several chronic diseases such as stroke and Parkinson^23–25^. In Western countries associations of DS with cancer and chronic diseases have been mostly evaluated in clinical studies which are biased towards more acutely ill individuals and have seldom been investigated in population-based samples, who may represent a less biased sample to gauge the prevalence of depressive symptoms^26,27^.

In the current study, using data from two population-based cohort studies, the Shanghai Women’s Health Study (SWHS) and the Shanghai Men’s Health Study (SMHS), we evaluate the prevalence of DS and the correlates of DS in a non-medical setting. To our knowledge, our study is one of the first to examine differences in the association of DS with chronic diseases by gender in a population-based setting, which enables a methodological advantage because psychological symptoms of those participants are the most prominent features, compared with patients in clinical study, who often present somatic complaints. Additionally, we examined whether DS was more likely to occur in an early stage of chronic disease (within 5 years of diagnosis) or a later stage of chronic disease (more than 5 years after diagnosis), to ascertain when risk for depression may be highest in the course of chronic disease in order to identify DS cases for early intervention. We also examine this issue in middle-aged and elderly adults in urban China, who represent a growing public health issue given that the proportion and number of elderly in China is set to increase rapidly so this contributes to a huge public health problem in China^9^.

## Research Design and Methods

### Participants

Participants of the SWHS and the SMHS, two population-based prospective studies, were included in this analysis. The detailed study designs of these two studies have been reported elsewhere^28,29^. Briefly, from typical urban communities in Shanghai, China, we recruited to the SWHS 74,940 women aged 40–70 years between 1996 and 2000 with a 92.7% participation rate, and to the SMHS, 61,478 men aged 40–74 years between 2002 and 2006 with a 74.0% participation rate. Reasons for non-participation in the SWHS and SMHS were refusal (SWHS: n = 2,408, 3.0%; SMHS: n = 17,823, 21.5%), being out of area during enrollment (SWHS: n = 2,073, 2.6%; SMHS: n = 2,370, 2.9%), and other miscellaneous reasons including poor health or hearing problems (SWHS: n = 1,748, 2.1%; SMHS: n = 1,360, 1.6%). In-person follow-up of all living cohort members was implemented from 2000 to 2002, 2002 to 2004, 2004 to 2007, and 2007–2011 with response rates of 99.8%, 98.7%, 95.0%, and 91.9% respectively in the SWHS, and from 2004 to 2008, and 2008 to 2011 with response rates of 97.7% and 91.9% in the SMHS. All survey interviews were conducted by trained staff using structured questionnaires. The studies were approved by the relevant institutional review boards for human research at the Shanghai Cancer Institute (China) and Vanderbilt University (United States) and all investigation methods were performed in accordance with relevant guidelines and regulations. Written, informed consent was obtained from all participants.

### Data collection

Structured questionnaires were used at the baseline and follow-up surveys to collect information on socio-demographics, behavior and lifestyles, history of diseases and anthropometric measurements. The follow-up surveys collected information on DS, survival status, and incidence of cancers and other selected chronic diseases and updated exposure information (such as dietary intake, weight, smoking and drinking, etc.).

### Depressive symptoms (DS)

At the fourth follow-up in the SWHS and the second follow-up in the SMHS, information on DS was collected using 7 questions below adapted from a validated Chinese version of the Center for Epidemiologic Studies Depression Scale (CES-D)^30^, which included the standard 20-item CES-D questions plus an additional 6 items specifically developed for Chinese participants. These questions were: (1) I felt that I could not get rid of depressed emotion even with help from my relatives and friends; (2) I felt I was in a blue mood; (3) I was happy; (4) I cried; (5) I felt I couldn’t go on with the routine of living; (6) I felt that so many words should be said, but I couldn’t find the appropriate chance to say them; (7) I felt that nobody could be trusted. The answer to each of these questions was the number of days in which the symptom was experienced in the past week, from 0 days to 7 days. While representing a shortened measure, these seven questions covered all four factors of the 20-item CES-D, which included depressed affect (question 1, 2 and 4 in current study), somatic retarded activity (question 5), interpersonal relations (question 6 and 7) and positive affect (question 3). The distribution of answers for question 3 (a positive item which was infrequently endorsed) suggested that this question may have been misinterpreted by the study participants because it was presented in the opposite direction of the other questions. Because this positive item appeared to be interpreted differently by our Chinese respondents and did not measure depressed mood in the same way as other items, it was excluded, thus acting to improve the reliability of the remaining questions^30^. According to a separate validation study of this shortened measure, we found it is comparable to use a 6 item instead of 26 item scale and to use DS score ≥4 as representing the cutoff for high DS in our analysis: (1) In a separate cohort study (Shanghai breast cancer survival study, SBCSS) of 5042 breast cancer survivors that was conducted in Shanghai China during the same study period as our DS survey in the SWHS and SMHS, we used the 26-item culturally-adapted and validated Chinese version of the CES-D in this survey. In this dataset: (a) We first calculated scores using all 26 items from Chinese version of the CES-D and all 20 items from standard CES-D, respectively, and (b) then separately, calculated subscale scores for the 6 items that we selected from Chinese version of the CES-D representing DS as per above. The correlation of our shortened 6-item scale with 20- and 26-item score was high (r = 0.81 and 0.84, respectively) and most variation (71%) in the 26-item score could be captured by the score in our shortened 6-item scale. (2) Using this initial validation from a separate sample as a basis, we evaluated the 6 identified questions in our current study. To evaluate reliability, we calculated Cronbach’s alpha of 0.78, which indicated that this scale had good reliability in this sample. (3) We assumed a unitary “depression factor” that could be measured by the score from our shortened 6-item scale and conducted a confirmatory factor analysis with the 6 items. Goodness-of-fit index was high (GFI = 0.95), which expresses that a single factor, overall depression construct, can capture most of variance among 6 items. (4) Individual scores were summed across each of the six questions to yield a total score from 0 to 42 in current study, with a higher score reflecting higher frequency of DS. Using the data in the SBCSS, We made a regression analysis of standard 20-item score on 6-item scale and found predicted value of 6-item scale was 3.5, given that the standard 20-item score = 16. Thus, following the CES-D score = 16 that indicates probable clinical depression^31^, this translated into a 6-item score = 4 (because our scale only counted whole integers), with a good AUC of 85.8% in current study. We conducted a sensitivity analysis using scores of 3, 4 or 5 as cut-off points to identify high DS for both men and women respectively and found data patterns to be similar no matter what cut-off point was used in analyses. Accordingly, we used scores = 4 to identify high DS for both men and women, which signifies risk for clinical depression. We applied the following three categories in the current study: 0 (no DS), DS score of 1–3 (some DS), and DS score ≥4 (high DS).

### Socio-demographics and lifestyle factors

Socio-demographic factors including gender, marital status, education, income and occupation assessed at the baseline survey for both women and men were used in current analysis. Age was recorded at the time of DS assessment. Information on lifestyle and BMI were collected at the baseline survey and updated during follow-up surveys prior to the DS assessment: data on ever smoking or drinking and amounts smoked and drank were collected at the baseline survey in both the SWHS and SMHS. Current smoking and drinking habits were evaluated in the fourth follow-up among women in the SWHS, while in men, current smoking data was collected in the first follow-up survey and current drinking was estimated in the baseline survey. Leisure-time physical activity was obtained from the third follow-up of the SWHS and from the baseline survey of the SMHS. BMI was calculated using the information from the baseline survey of the SMHS and the third follow-up of the SWHS (please see footnotes of tables for detailed information of time windows in data collection).

### Diseases

Information on occurrence of cancers and chronic diseases such as diabetes, hypertension, myocardial infarction, stroke, Parkinson’s disease, and bone fracture was collected at baseline and updated in the follow-up surveys. Only disease cases diagnosed before DS assessment were included in the current analysis.

### Statistical analysis

This study included 103,595 participants, 50,006 from the SMHS and 53,589 from the SWHS who participated in the DS survey. In the analysis, age at the time of DS assessment was grouped into three categories: < 60 years, 60–69 years, and ≥70 years. Other factors included in the analysis were: marital status (married, widowed and other (divorced/single/separated)), educational level (elementary school or less, middle school, high school, and college or above), annual per capita income (<6000, 6000- and ≥10000 yuan in the SMHS and <4000, 4000- and ≥8000 yuan in the SWHS), job held longest (manual worker, clerical, or professional), and smoking status (non-smokers, past smokers and current smokers). The quantity of smoking was categorized into 3 groups: non-smokers, <35 packs/year, and ≥35 packs/year. Alcohol drinkers were grouped into three categories: non-drinkers, past drinkers, and current drinkers. The quantity of alcohol consumed was classified into 3 categories: non-drinkers, <1.0 drinks/day, and ≥1.0 drinks/day. BMI was calculated as weight in kilograms divided by the square of height in meters and was divided into four groups: <18.5, 18.5–24.9 (reference), 25–29.9 and ≥30. Leisure-time physical activity was presented as a dichotomous variable (yes/no).

One-way ANOVA (Wilcoxon test for skewed data) or *χ*^2^ test was applied to evaluate differences between individuals with and without DS. Using the absence of DS as a reference, a polynomial logistic regression model was used to calculate odds ratios (ORs) and their 95% confidence intervals (95% CIs) for a low depression score (DS score = 1–3) and high depression score (DS score                    ≥ 4) associated (no DS as reference) with age, gender, marital status, education level, occupational status and individual income with mutual adjustments. A trend test was carried out by entering the categorical variables as continuous parameters in the models. We analyzed the associations between DS and lifestyle factors, adjusted for the socio-demographic factors mentioned above, and analyzed the associations between DS and cancer and other chronic diseases adjusted for both socio-demographic and lifestyle factors. All analyses were carried out using Statistical Analysis System (SAS, version 9.4; SAS Institute, Cary, NC). The significance level was *P* ≤ 0.05 based on two-sided tests.

Researchers who wish to gain access to the data used in this study can visit our website (https://www.mc.vanderbilt.edu/swhs-smhs/index.html) and submit an application according to the data & biological sample guidelines of the Shanghai Men’s Health Study and Shanghai Women’s Health Study.

## Results

The average age of participants at the time of the depression survey was 61.8 years (standard deviation: 9.3 years).The overall prevalence of DS was 4.1%. Prevalence was lower in men than women (2.4% in men and 5.6% in women, *P* < 0.01). Of participants who reported DS, 60.5% reported one symptom, 21.6% reported two symptoms, 8.5% reported three, and 9.4% reported more than three symptoms.

Table 1 shows differences in socio-demographics, lifestyle factors, and health conditions between individuals with and without DS (DS counts all people with DS score ≥1). Both men and women with DS were older, had lower incomes and BMI, and had a higher prevalence of chronic diseases or cancer than those who reported no DS. Women were also more likely to report DS if they were current smokers, not married, were manual workers, or had a lower education level. These correlations, however, were not observed in men.

**Table 1:** Socio-demographic characteristics and lifestyle factors of participants in the SMHS and SWHS.

	Total (N = 103595)	Male			Female		
		No DS* (N = 48783)	DS* (N = 1223)	P value	No DS* (N = 50607)	DS* (N = 2982)	P value
**Age** (years, $\bar{x}$± *SD*)^b^	61.8 ± 9.3	60.9 ± 9.6	62.5 ± 10.1	<0.0001	62.6 ± 8.9	63.9 ± 9.2	<0.0001
**Age**(%)^b^							
<60	52708 (50.9)	26598 (97.9)	582 (2.1)	<0.0001	24233 (94.9)	1295 (5.1)	<0.0001
60-	25062 (24.2)	11086 (97.6)	274 (2.4)		12994 (94.8)	708 (5.2)	
≥70	25825 (24.9)	11099 (96.8)	367 (3.2)		13380 (93.2)	979 (6.8)	
**Marriage**(%)^a^							
Married	90836 (93.1)	41694 (97.6)	1039 (2.4)	0.9737	45497 (94.6)	2606 (5.4)	<0.0001
Widowed	3905 (4.0)	244 (97.6)	6 (2.4)		3404 (93.1)	251 (6.9)	
Others	2781 (2.9)	928 (97.7)	22 (2.3)		1706 (93.2)	125 (6.8)	
**Education**(%)^a^							
Elementary or under	14537 (14.0)	3074 (97.1)	91 (2.9)	0.1818	10584 (93.1)	788 (6.9)	<0.0001
Middle or high school	70654 (68.2)	34231 (97.5)	861 (2.5)		33663 (94.7)	1899 (5.3)	
College or above	18404 (17.8)	11478 (97.7)	271 (2.3)		6360 (95.6)	295 (4.4)	
**Income (RMB/person/year**, %**)**^a,**^							
<6000(4000)	14829 (14.3)	6111 (97.1)	180 (2.9)	0.0004	7941 (93.0)	597 (7.0)	<0.0001
6000(4000)-	79245 (76.6)	37915 (97.5)	960 (2.5)		38180 (94.6)	2190 (5.4)	
≥10000(8000)	9418 (9.1)	4663 (98.3)	81 (1.7)		4479 (95.8)	195 (4.2)	
**Occupation(**%)^a^							
Manual worker	53831 (52.0)	25341 (97.5)	658 (2.5)	0.2195	26154 (94.0)	1678 (6.0)	<0.0001
Clerical	22006 (21.2)	10638 (97.8)	242 (2.2)		10528 (94.6)	598 (5.4)	
Professional	27702 (26.8)	12750 (97.5)	321 (2.5)		13925 (95.2)	706 (4.8)	
**BMI**(kg/m^2^, %)^a,b^							
<18.5	3420 (3.3)	1418 (95.1)	73 (4.9)	<0.0001	1766 (91.6)	163 (8.4)	<0.0001
18.5-	64212 (62.0)	30444 (97.6)	752 (2.4)		31254 (94.7)	1762 (5.3)	
25-	32091 (31.0)	15499 (97.7)	370 (2.3)		15302 (94.3)	920 (5.7)	
≥30	3872 (3.7)	1422 (98.1)	28 (1.9)		2285 (94.3)	137 (5.7)	
**Leisure-time physical activity** ^d^							
No	64336 (62.1)	31554 (97.7)	750 (2.3)	0.0153	30210 (94.3)	1822 (5.7)	0.1285
Yes	39259 (37.9)	17229 (97.3)	473 (2.7)		20397 (94.6)	1160 (5.4)	
**Current smokers** (%)^c^							
No	70418 (72.8)	17458 (97.4)	471 (2.6)	0.0919	49596 (94.5)	2893 (5.5)	0.0002
Yes	26238 (27.2)	24542 (97.6)	596 (2.4)		1011 (91.9)	89 (8.1)	
**Current drinkers** (%)^d^							
No	86504 (83.5)	34364 (97.5)	877 (2.5)	0.3376	48389 (94.4)	2874 (5.6)	0.0475
Yes	17091 (16.5)	14419 (97.7)	346 (2.3)		2218 (95.4)	108 (4.6)	
**Diseases**(%)^e^							
No	38992 (37.6)	16398 (98.1)	319 (1.9)	<0.0001	21305 (95.6)	970 (4.4)	<0.0001
Yes	64603 (62.4)	32385 (97.3)	904 (2.7)		29302 (93.6)	2012 (6.4)	

*DS: Depressive symptoms. **Cut points for females in brackets.

^a^Data from baseline survey for both SMHS and SWHS.

^b^Data from the second follow-up of the SMHS and the fourth follow-up of the SWHS.

^c^Data from the first follow-up of the SMHS and the fourth follow-up of the SWHS.

^d^Data from the baseline survey of the SMHS and the fourth follow-up of the SWHS.

^e^Data from the baseline survey and all follow-ups in both the SMHS and the SWHS. Diseases include diabetes, hypertension, stroke, myocardial infarction, bone fracture, Parkinson’s, cataract, hepatitis, prostate, and cancer. All these diseases were diagnosed before the depressive symptom survey.

Table 2 presents odds ratios of DS categories in association with socio-demographic factors by gender. Age was significantly associated with DS in both men and women. The ORs of having some and high DS increased about 1.6 (95% CI: 1.3–1.9) and 1.7 (95% CI: 1.3–2.2) times for men aged ≥70 years; and 1.2 (95% CI: 1.0–1.4) and 1.5 (95% CI: 1.2–1.8) times for women aged ≥70 years, compared to those <60 years. Significant associations with other socio-demographic factors were observed only in women: being unmarried (including divorced, single and separated) was associated with high DS (OR = 1.3, 95% CI: 1.0–1.8), while a high education level, high income were associated with 17%- 34% reduced odds ratio of having a high DS, respectively.

**Table 2:** Associations of socio-demographic characteristics and depressive symptoms by gender**.

	Male		Female	
	Some DS (N = 845)	High DS (N = 378)	Some DS (N = 1817)	High DS (N = 1165)
**Age** (years)				
<60	1.0	1.0	1.0	1.0
60-	1.15 (0.96–1.38)	1.35 (1.03–1.76)	0.98 (0.87–1.13)	1.18 (1.01–1.38)
≥70	1.59 (1.33–1.90)	1.71 (1.31–2.24)	1.17 (1.01–1.35)	1.47 (1.23–1.76)
*P* for trend	<0.0001	<0.0001	0.1076	<0.0001
**Marriage**				
Married	1.0	1.0	1.0	1.0
Widowed	0.39 (0.10–1.59)	1.77 (0.65–4.80)	0.95 (0.79–1.15)	1.18 (0.96–1.45)
Others	0.88 (0.52–1.51)	1.23 (0.60–2.49)	1.23 (0.97–1.56)	1.33 (1.00–1.79)
**Education**				
Elementary or under	1.0	1.0	1.0	1.0
Middle or high school	1.15 (0.87–1.53)	1.07 (0.71–1.63)	1.05 (0.90–1.22)	0.84 (0.71–1.01)
College or above	1.03 (0.74–1.45)	1.19 (0.73–1.93)	0.98 (0.79–1.22)	0.66 (0.50–0.87)
*P* for trend	0.7202	0.4232	0.7439	0.0024
**Occupation**				
Manual worker	1.0	1.0	1.0	1.0
Clerical	0.95 (0.79–1.13)	0.77 (0.58–1.02)	0.92 (0.81–1.04)	0.95 (0.82–1.11)
Professional	0.92 (0.75–1.13)	1.00 (0.74–1.34)	0.89 (0.78–1.02)	0.88 (0.74–1.05)
Income(RMB/person/year)*				
<6000(4000)	1.0	1.0	1.0	1.0
6000-(4000-)	0.80 (0.65–0.97)	0.75 (0.56–1.02)	0.82 (0.72–0.93)	0.87 (0.75–1.01)
≥10000(8000)	0.53 (0.38–0.75)	0.65 (0.40–1.04)	0.73 (0.58–0.90)	0.68 (0.51–0.91)
*P* for trend	0.0001	0.0695	0.0004	0.0075

*Cut points in brackets are for females.

**Low and high depression score represent that participants had depressive symptoms in 1–3 days and ≥4 days, respectively. Age, marriage, education level, occupation and income were mutually adjusted in the polynomial logistic regression.

The associations between DS and lifestyle factors are presented in Table 3. Having a low BMI was significantly associated with DS in both men and women. ORs associated with BMI of less than 18.5 were 1.8 (95% CI: 1.4–2.5) and 2.2 (95% CI: 1.5–3.3) for some and high DS in men and 1.5 (95% CI: 1.2–1.8) and 1.7 (95% CI: 1.3–2.2) for some and high DS in women, respectively, compared those with normal BMI (18.5–24.9). In female smokers, both currently smoking and amount smoked (packs/year) were associated with an over 1.5 times higher likelihood of having high DS. Leisure-time physical activity was inversely associated with high DS in women (OR = 0.8; 95% CI: 0.7–0.9), but not in men. We did not find alcohol consumption to be associated with DS in either men or women.

**Table 3:** Association of lifestyle factors and depressive symptoms by gender*.

	Male			Female		
	N	Some DS (N = 845)	High DS (N = 378)	N	Some DS (N = 1817)	High DS (N = 1165)
**Smoking**						
No	14504	1.0	1.0	51803	1.0	1.0
Past	10364	1.15 (0.94–1.39)	1.25 (0.94–1.65)	686	1.04 (0.70–1.55)	1.39 (0.93–2.08)
Current^c^	25138	1.11 (0.93–1.32)	1.06 (0.82–1.36)	1100	1.19 (0.88–1.61)	1.61 (1.18–2.20)
**Quantity of smoking** (Pack/year)^a^						
No	15204	1.0	1.0	52167	1.0	1.0
<35	23889	1.18 (0.97–1.44)	1.00 (0.75–1.34)	704	1.07 (0.72–1.59)	1.40 (0.91–2.15)
≥35	10913	1.04 (0.86–1.26)	1.17 (0.89–1.53)	718	1.27 (0.89–1.81)	1.45 (1.00–2.09)
*P* for trend		0.5096	0.2964		0.1841	0.0172
**Alcohol consumption**						
No	33110	1.0	1.0	50500	1.0	1.0
Past	2131	1.19 (0.88–1.63)	1.70 (1.15–2.53)	763	0.74 (0.47–1.16)	1.02 (0.64–1.63)
Current^d^	14765	1.00 (0.85–1.16)	0.94 (0.74–1.18)	2326	0.76 (0.59–1.00)	0.88 (0.66–1.19)
**Quantity of drinking**(drink/day)^a^						
No	33110	1.0	1.0	52498	1.0	1.0
<1.00	3327	0.88 (0.66–1.18)	0.74 (0.47–1.19)	854	0.74 (0.48–1.13)	0.66 (0.38–1.15)
≥1.00	13326	1.06 (0.91–1.24)	1.11 (0.88–1.39)	237	0.83 (0.39–1.76)	1.52 (0.78–2.98)
*P* for trend		0.5476	0.4821		0.1910	0.9550
**BMI(**kg/m^**2**^**)**^**a,b**^						
<18.5	1491	1.83 (1.35–2.48)	2.22 (1.47–3.34)	1929	1.48 (1.20–1.84)	1.71 (1.33–2.21)
18.5-	31196	1.0	1.0	33016	1.0	1.0
25-	15869	0.96 (0.83–1.12)	0.96 (0.77–1.20)	16222	0.96 (0.86–1.07)	1.12 (0.98–1.27)
≥30	1450	0.88 (0.57–1.35)	0.55 (0.24–1.23)	2422	0.98 (0.78–1.23)	0.94 (0.71–1.25)
*P* for trend		0.0196	0.0083		0.0286	0.3682
**Leisure-time physical activity** ^**e**^						
No	32304	1.0	1.0	32032	1.0	1.0
Yes	17702	1.06 (0.91–1.24)	0.95 (0.76–1.20)	21557	0.92 (0.83–1.01)	0.84 (0.74–0.95)

*Using polynomial logistic regression adjusted for age (continuous), marriage, income, occupation and education.

^**a**^Data from baseline survey of both SMHS and SWHS.

^**b**^Data from the second follow-up of the SMHS and the fourth follow-up of the SWHS.

^**c**^Data from the first follow-up of the SMHS and the fourth follow-up of the SWHS.

^**d**^Data from the baseline survey of the SMHS and the fourth follow-up of the SWHS.

^**e**^Data from the baseline survey of the SMHS and the third follow-up of the SWHS.

Table 4 presents associations of cancer and selected chronic diseases with prevalence of DS stratified by interval (in 5 year increments) between disease diagnosis and initial DS assessment. The prevalence of DS in Parkinson’s patients was 8.5% (4.3% for men and 11.8% for women), the highest among all individuals with chronic diseases evaluated in the current study. Parkinson’s disease was significantly associated with high DS in both men and women, with ORs of 3.1 (95% CI: 1.3–7.7) in men and 2.7 (95% CI: 1.6–4.7) in women, but this association was only significant in women more than 5 years after their Parkinson’s diagnosis, while in men, this association was only significant among those who were within 5 years post-diagnosis. Stroke was significantly associated with some and high DS, with OR ranged from 1.5 to 2.2 for both men and women regardless of the interval between stroke diagnosis and the depression assessment. Similarly, cancer was associated DS (OR = 1.5; 95% CI: 1.0–2.3 for some DS and OR = 3.3; 95% CI: 2.2–5.0 for high DS) in men, and (OR = 1.7; 95% CI: 1.2–2.3 for some DS and OR = 1.9; 95% CI: 1.4–2.7 for high DS) in women. In men, these associations were observed among survivors whose cancer was diagnosed within 5 years of the cancer diagnosis. But in women, these associations were significant, with the exception of some DS among those who were more than 5 years post-cancer diagnosis. Other chronic diseases, including hypertension, cataracts, bone fracture, myocardial infarction and prostatic disease were associated with DS, with ORs of 1.1–2.2. We found no significant associations between diabetes (defined according the criteria of American Diabetes Association), hepatitis and DS.

**Table 4:** Association of diseases and depressive symptoms by gender and by 5-year increment between diagnosis and depression survey*.

	Male				Femae			
	Total	DS	Some DS	High DS	Total	DS	Some DS	High DS
**Stroke**								
No	46421	1064	1.0	1.0	49553	2614	1.0	1.0
Yes	3585	159	1.51 (1.20–1.89)	2.18 (1.62–2.94)	4036	368	1.52 (1.30–1.79)	1.76 (1.47–2.09)
<5 years	1798	88	1.89 (1.44–2.49)	1.99 (1.32–2.99)	2323	216	1.57 (1.29–1.91)	1.82 (1.46–2.26)
≥5 years	1555	65	1.21 (0.85–1.73)	2.33 (1.56–3.49)	1673	151	1.49 (1.18–1.88)	1.71 (1.32–2.21)
**Myocardial infarction**								
No	49186	1191	1.0	1.0	53201	2950	1.0	1.0
Yes	820	32	1.75 (1.17–2.62)	0.82 (0.36–1.85)	388	32	1.74(1.13–2.66)	0.91(0.47–1.78)
<5 years	412	18	2.21 (1.33–3.67)	0.59 (0.15–2.39)	242	24	2.07(1.26–3.40)	1.13(0.53–2.40)
≥5 years	408	14	1.31 (0.69–2.48)	1.00 (0.37–2.73)	138	8	1.26(0.56–2.87)	0.58(0.14–2.36)
**Diabetes**								
No	45267	1079	1.0	1.0	48102	2617	1.0	1.0
Yes	4739	144	1.18 (0.95–1.47)	1.31 (0.96–1.78)	5487	365	1.12 (0.97–1.30)	1.16 (0.97–1.38)
<5 years	1578	42	1.07 (0.73–1.57)	1.24 (0.72–2.12)	1618	109	1.25 (0.98–1.61)	1.11 (0.81–1.53)
≥5 years	3156	100	1.21 (0.94–1.57)	1.32 (0.91–1.90)	3862	255	1.07 (0.89–1.28)	1.17 (0.95–1.43)
**Hypertension**								
No	32545	705	1.0	1.0	33876	1776	1.0	1.0
Yes	17461	518	1.34 (1.16–1.56)	1.37 (1.10–1.71)	19713	1206	1.10 (0.99–1.22)	1.16 (1.02–1.31)
<5 years	2472	81	1.41 (1.05–1.89)	1.92 (1.30–2.82)	2887	174	1.10 (0.90–1.36)	1.22 (0.95–1.56)
≥5 years	14980	436	1.33 (1.14–1.55)	1.26 (1.00–1.59)	16566	1009	1.10 (0.98–1.22)	1.12 (0.98–1.27)
**Cancer**								
No	49086	1172	1.0	1.0	52775	2905	1.0	1.0
Yes	920	51	1.54 (1.04–2.30)	3.31 (2.18–5.03)	814	77	1.66 (1.22–2.26)	1.92 (1.35–2.74)
<5 years	849	50	1.61 (1.07–2.42)	3.60 (2.37–5.48)	538	57	2.10 (1.49–2.95)	1.91 (1.23–2.98)
≥5 years	71	1	—	—	276	20	0.86 (0.42–1.74)	1.93 (1.08–3.46)
**Parkinson’s**								
No	49844	1216	1.0	1.0	53385	2958	1.0	1.0
Yes	162	7	0.62 (0.15–2.53)	3.13 (1.26–7.75)	204	24	1.45 (0.77–2.75)	2.71 (1.56–4.71)
<5 years	72	5	1.51 (0.37–6.19)	4.65 (1.44–14.9)	93	7	0.61 (0.15–2.50)	2.04 (0.82–5.06)
≥5 years	89	2	—	—	110	17	2.23 (1.08–4.62)	3.35 (1.68–6.70)
**Hepatitis**								
No	44478	1089	1.0	1.0	52075	2881	1.0	1.0
Yes	5528	134	0.87 (0.69–1.10)	1.34 (1.00–1.80)	1514	101	1.18 (0.91–1.54)	1.33 (0.97–1.82)
<5 years	144	6	0.86 (0.21–3.47)	3.90 (1.42–10.7)	111	4	0.54 (0.13–2.19)	0.88 (0.22–3.58)
≥5 years	5376	128	0.87 (0.69–1.10)	1.28 (0.94–1.73)	1398	97	1.24 (0.94–1.62)	1.37 (0.99–1.89)
**Cataract**								
No	44688	1029	1.0	1.0	42172	2106		
Yes	5318	194	1.38 (1.12–1.70)	1.40 (1.04–1.89)	11417	876	1.53 (1.35–1.72)	1.50 (1.30–1.73)
<5 years	2312	92	1.52 (1.15–2.01)	1.59 (1.08–2.35)	4461	336	1.44 (1.22–1.69)	1.63 (1.35–1.96)
≥5 years	2968	100	1.25 (0.95–1.64)	1.22 (0.82–1.81)	6797	532	1.61 (1.40–1.86)	1.35 (1.14–1.61)
**Bone fracture**								
No	35588	849	1.0	1.0	46184	2491	1.0	1.0
Yes	14418	374	1.01 (0.87–1.18)	1.26 (1.02–1.57)	7405	491	1.16 (1.02–1.32)	1.28 (1.10–1.50)
<5 years	1014	32	0.99 (0.61–1.61)	2.07 (1.23–3.51)	3444	255	1.31 (1.10–1.56)	1.46 (1.19–1.79)
≥5 years	13396	342	1.02 (0.87–1.18)	1.20 (0.96–1.50)	3710	220	1.04 (0.86–1.25)	1.11 (0.90–1.39)
**Prostatic disease**								
No	47544	1114	1.0	1.0				
Yes	2462	109	1.67 (1.30–2.16)	2.21 (1.58–3.08)				
<5 years	2070	93	1.76 (1.34–2.30)	2.11 (1.46–3.04)				
≥5 years	372	13	1.13 (0.53–2.39)	2.10 (0.93–4.76)				

*Using polynomial logistic regression adjusted for age (continuous), marriage, income, education, occupation, smoking, drinking, BMI and leisure-time physical activity.

## Discussion

In this large population-based study of men and women of age 43–84 years (average of 60.9 and 62.7 years for men and women respectively) in urban Shanghai, we found a prevalence of DS of 4.1%, a rate that is lower than some recent reports from cities of west China and rural areas^11,15,32^. However, given the continuing stressors from rapid economic growth and urbanization, the prevalence of DS in urban China is likely to increase in the future. Our findings represent an important contribution to public health in China, given that understanding the mental health needs of the elderly is crucial due to their rapidly-expanding numbers^33,34^. Our finding is consistent with another report which found the exact same prevalence of DS (4.1%) among middle and elderly residents of Shanghai but a much higher prevalence rate among people living in Beijing (14.9%)^14^. While the reason underlying the vast difference in DS prevalence between these two major Chinese cities is unknown, differences in lifestyle, dietary habit, living environment and prevalence of chronic diseases may be one of the attributes. We found that prevalence of DS was twice as high in women as in men, similar to findings from a larger number of studies using similar assessments^17,18^ as well as recent reports on major depression disorder^35^. In addition, old age was positively associated with DS, while both income and BMI were inversely associated with DS. In women, having a professional occupation, a high education level, and exercise regularly were all associated with reduced prevalence of DS, while being unmarried or currently smoking was significantly associated with increased prevalence. Cancer and several other chronic diseases, including stroke, Parkinson’s disease, hypertension, cataracts, prostatic diseases, as well as bone fracture, were significantly associated with increased prevalence of DS in both men and women.

Our finding that aging was associated with DS in both men and women is consistent with several previous reports^12,36,37^. Aging is characterized by important biological, psychological and social changes, and thus may affect DS prevalence. The association could also result from changes that often come in later life, including retirement, the death of loved ones, increased isolation, and medical problems^37^. Due to the one-child policy, many elderly people may be forced to live alone or with their spouse only; further, family support from younger generations is shrinking, all of which could be important mechanisms of DS in the elderly^38^. Social determinants (such as education, income, occupation and gender) are important factors in the development of DS. Women are nearly twice as likely as men to exhibit DS in our study. The reasons for this are multifactorial and include biological and environmental factors, including but not limited to: a longer life expectancy and a higher widowhood rate, work overload, and unequal power and status for women in China^9^. Another biological factor includes sex hormones that play an important role in gender differences in the prevalence of DS^39,40^. Income, a strong indicator of current socioeconomic position, was reported to be associated with reduced DS in many studies^14,17,18^. Our study confirmed this association. Education, an important contributing factor for socioeconomic status, has often been linked DS with advanced education being associated with low prevalence of DS^10,14,32,41^. Education, as a source of human capital, may motivate and facilitate individuals in pursuing fundamental outcomes that include emotional well-being^42^, and it also precedes and influences other socioeconomic factors such as income and occupation that are themselves related to DS. However, in our study, this protective effect of education on DS was observed only in women because women with a high education level and income may be better endowed with resources, such as coping style, self-esteem, mastery, and locus of control, that could buffer the impact of stress-induced DS^43^ Other factors, including geography and urbanization, are found to be important factors in DS development, especially in China because from 2012 more than half of its population live and work in urban areas and there has been massive rural-to-urban migration to Chinese cities^44^. In a 2011 Migration and Quality of Life Survey, Chen *et al*. reported highly populated cities along the eastern coast such as Shanghai illustrated potential effects of urbanization as a stressor for mental health^45^.

In a Hong Kong study, Li *et al*. found that underweight individuals had a high prevalence of DS and overweight ones a low prevalence, compared to those in the normal weight range^46^. Yu *et al*. found that thin men had a higher prevalence of DS than normal weight men in Taiwan^47^. In rural China, obese men were found to be less likely to suffer from DS, compared with normal weight men^48^. Consistent with these Chinese studies, we found that BMI was negatively associated with prevalence of DS in men and in women. However, in two meta-analysis studies, one for cohort studies and another for cross-sectional studies, mainly conducted in Western populations, reported a significantly positive association between DS and obesity^49,50^. A United States-based study reported that major depressive symptoms were associated with a low BMI in men but a high BMI in women^22^. A study in the Netherlands observed a significant U-shaped association between BMI (underweight, normal, overweight and obesity) and DS, i.e., both obesity and underweight were associated with an increased risk of DS^51^. The underlying mechanism for this discrepancy is not entirely clear. Among middle-aged and elderly Chinese people, the prevalence of obesity is low (3.7% in this study) and severe obesity is rare; the association pattern might be explained by the ‘happy mind and fat body’ culture because many overweight or modestly obese people tend to be more optimistic and happier than those who are thinner in China^21,46^. But in Western countries, obesity, particularly morbid obesity, could be related to greater stress, stigma, chronic diseases and devaluation, causing obese people to suffer lower self-esteem and have a more negative self-image, resulting in more DS^49^. Although several studies reported that BMI or obesity is inversely correlated with DS, some of them only observed the impact of DS on BMI and did not observe an inverse effect of BMI on DS as we did in our study. This directionality could lead to a different conclusion. Forman-Hoffman *et al*. reported that the effects of DS on weight differ by gender, and concluded that DS at baseline may predict both weight loss and weight gain^52^. However, Konttinen *et al*. reported that men with depressive symptoms were likely to have a higher BMI, while women with a higher BMI were likely to develop depressive symptoms^53^. In middle aged and elderly Asian populations, depression can lead to weight loss rather than obesity, and being underweight may elicit DS unlike among Westerners who show a positive relationship between depression and obesity. These findings have implications for public health interventions by gender in China^54^.

Groffen *et al*. reported that only current smoking, not past smoking, was associated with DS in white women in US^55^. Current smoking was associated with increased prevalence of DS in the Korean and Japanese female populations^56,57^. On the other hand, a study in Eastern Europe found that current and past smoking was associated with DS in men but not in women^58^. Effects of smoking on DS are complex: Nicotine has antidepressant properties, releasing dopamine in the mesolimbic reward pathway, thereby elevating mood and relieving stress^59^. However, evidence has also suggested that smoking increases a person’s risk of DS as a result of changes in neurophysiology^60^. In our study, ever smoking rate in men was 71% (50.3% for current smoking, 20.7% for past smoking), much higher than in many western countries, but the prevalence of DS was low (2.4%). We found no statistical difference in DS between smokers and non-smokers among men in our study. However, smoking rate was low in women (2.0%) in our study, and female current smokers had a high prevalence DS compared with non-smokers. It is unclear whether such association was caused by women with DS seeking stress relief via smoking. An alternative explanation is that smoking was not socially acceptable for women, especially in urban China. Thus, female smokers could have low self-esteem which led to an increase of DS^57,61^.

Another contribution was that our study assessed whether DS was more likely to occur in an early vs. late stage of chronic disease (i.e., within 5 years of diagnosis, or after this period) to identify when risk for depression is most elevated. Depression is a significant problem for cancer patients in particular, either as a pre-existing or co-existing condition, or as a consequence of cancer diagnosis and treatment. Several studies have reported that the associations of cancer with DS lasted for varying lengths of time depending on the type of cancer^23,62^. However, few studies have focused on assessing DS among long-term cancer survivors in a non-medical setting^62,63^. In our population-based study, we found an increased DS prevalence primarily within 5 years post-cancer diagnosis. This association persisted but was attenuated when the time interval since diagnosis increased to beyond 5 years in women. These findings suggest a need of proper intervention and care for mental health for cancer survivors, particularly during the period soon after cancer diagnosis. Consistent with previous findings^24,25^, we found that patients with Parkinson’s disease or stoke had an increased prevalence of DS that appeared relatively consistent both before and after 5 years post-diagnosis. These associations call for a long term management/treatment approach.

Our study has many strengths, including a large sample size, a population-based design, and adjustment for a wide range of socioeconomic characteristics, lifestyle factors, chronic diseases, and cancer. Another noticeable strength is that the measure of all physical illnesses took place prior to the CES-D measurement, minimizing the risk of reverse causation. However, several limitations of our study should also be acknowledged. (1) The relatively low participation rate of 74.0% among men in the SMHS, compared with that of women in the SWHS, could lead to selection bias in parameter estimation. Reasons for non-participation included refusal, being out of the area during enrollment and other miscellaneous reasons, including health and hearing problems. However, lower participation of men is consistent with other large psychiatric epidemiology studies^64^, including those conducted in China^65^. A study from the Danish National Birth Cohort reported that in a cohort study based on prospective data, the decision to participate cannot be based on future outcomes; this suggests a minimal risk of bias in our study given that DS outcomes were collected prospectively^66^. (2) We used data from two large cohort studies: the SWHS and the SMHS. However, these datasets did not allow examination of some key risk factors, including but not limited to early childhood adversity and psychosocial stress at work. (3) We only included 6 CES-D questions instead of the full 20- or 26-item measure in the study, which may not have captured all possible DS and therefore could have resulted in an underestimate of the prevalence of DS in our study population. Further, the CES-D indicates the likelihood of clinical depression, not clinical depression itself. Our study would have been strengthened if our shortened DS measure was validated by a sub-sample where clinical interviews were conducted to evaluate our shortened measure’s ability to accurately detect clinical depression. However, these concerns are at least somewhat mitigated by the psychometric analyses that we conducted above on our 6-item measure. (4) Our study was conducted among long-term urban residents in Shanghai, one of the most developed cities in China. Thus, the results of our study may not be generalizable to all Chinese, particularly those living in rural areas or those who migrated to urban areas.

In conclusion, in this large population-based study of 103,595 Chinese women and men, we found that 2.4% and 5.6% of middle-aged and elderly Chinese men and women living in Shanghai experienced DS. Age and several socio-demographic characteristics and lifestyle factors, such as education, income, smoking, cancer and several chronic diseases were associated with DS. In future studies, qualitative approaches would be quite valuable in further elucidating the nature of DS among elderly, and whether these are tied to perceived changes in aging, health status, socioeconomic status, or cultural standing in relation to modernization. Findings of our study are value for development of prevention programs in identifying elderly individuals with DS for early intervention.
